# *In vitro* antimicrobial activity on clinical microbial strains and antioxidant properties of *Artemisia parviflora*

**DOI:** 10.1186/1476-0711-11-30

**Published:** 2012-11-21

**Authors:** Abdul R Ahameethunisa, Waheeta Hopper

**Affiliations:** 1 Department of Bioinformatics, School of Bioengineering, Faculty of Engineering & Technology, SRM University, Kattankulathur, Tamil Nadu 603203, India

**Keywords:** *Artemisia*, Terpenoids, Antimicrobial, Antioxidant, Radical scavenging activity

## Abstract

**Background:**

*Artemisia parviflora* leaf extracts were evaluated for potential antimicrobial and antioxidant properties. Antimicrobial susceptibility assay was performed against ten standard reference bacterial strains. Antioxidant activity was analyzed using the ferric thiocyanate and 2, 2-Diphenyl-1-Picrylhydrazyl (DPPH) assays. Radical scavenging activity and total phenolic content were compared. Phytochemical analyses were performed to identify the major bioactive constitution of the plant extract.

**Results:**

Hexane, methanol and ethyl acetate extracts of *A. parviflora* leaves exhibited good activity against the microorganisms tested. The n-hexane extract of *A. parviflora* showed high inhibition of the growth of *Pseudomonas aeruginosa, Escherichia coli* and *Shigella flexneri*. Methanol extract showed strong radical scavenging and antioxidant activity, other extracts showed moderate antioxidant activity. The major derivatives present in the extracts are of terpenes, steroids, phenols, flavonoids, tannins and volatile oil.

**Conclusions:**

The results obtained with n-hexane extract were particularly significant as it strongly inhibited the growth of *P. aeruginosa, E. coli* and *S. flexneri*. The major constituent of the n-hexane extract was identified as terpenes. Strong antioxidant activity could be observed with all the individual extracts. The antimicrobial and antioxidant property of the extracts were attributed to the secondary metabolites, terpenes and phenolic compounds present in *A. parviflora* and could be of considerable interest in the development of new drugs.

## Background

*Artemisia* belonging to the family Asteraceae is known for their essential oils. More than 400 species of *Artemisia* have been explored from different parts of the world. The strong aroma and bitter taste of *Artemisia* are from the terpenoids and sesquiterpene lactones, constituents of the essential oil [1]. Essential oils of *Artemisia* spp. have been widely used for a variety of medicinal purposes such as antimalarial, antibacterial, antiviral, nematicidal and fungicidal for many years [2-7]. *A. parviflora* is an aromatic shrub, about 30–90 cm in height, found throughout the greater part of India at altitudes of 900–3000 m [8]. *A. parviflora* yields a light yellow essential oil which contains isobornyl acetate (7.2%), ar-curcumene (4.7%), limonene (1.6%), propanol (1.2%) as the identified components and an unidentified portion (77.9%). Later from the unidentified portion of *A. parviflora* essential oil, Β- caryophyllene, germacrene D, camphor and artemisia ketone were identified as the major constituents [9].

Antibiotic resistance by microorganisms causing infectious diseases has become a global concern in recent years. The worldwide emergence of multi-drug resistant *Escherichia coli, Klebsiella pneumoniae, Pseudomonas aeruginosa* and many other ß-lactamase producers has become a major therapeutic problem. Plants are valuable sources of natural products. The use of compounds from *Artemisia* spp. for pharmaceutical purposes has gradually increased [10-15]. Antimicrobial activity by *Artemisia* spp. against multi-drug resistant strains of *Bacillus subtilis, Staphylococcus aureus, E. coli*, *K. pneumoniae* and *P. aeruginosa,* opportunistic pathogens widely distributed in hospitals as well as increasingly being isolated from community acquired infections, have been reported [16-18]*.*

Knowledge of the chemical constituents of plants is desirable, not only for the discovery of therapeutic agents, but also because such information may be of value in disclosing new sources. Conventionally available synthetic antibacterial and antioxidant drugs are often associated with undesirable side effects and antibacterial drug resistance problem. The use of phytochemicals with known antimicrobial properties can be of great significance in therapeutic treatment of resistant strains and clinical pathogens. *Artemisia* species were reported for high content of phenolic and flavonoids which possess strong antioxidant properties and radical scavenging activities [19-21]. In this study, the antimicrobial activity of various extracts of *A. parviflora* was studied against clinical pathogens. The phytochemical composition and the assessment of the antioxidant potential of the crude extracts were also tested.

## Methods

### Plant material and extraction

The fresh plant of *Artemisia parviflora* was collected from Tropical Botanical Gardens, Trivandrum, Kerala. The plant specimen was authenticated and voucher specimen (SRMU/BI/4) was deposited in the Herbarium at Proteomics lab, SRM University. The leaves were dried under shade. After drying for a week, the leaves were powdered and sieved using gauze cloth. The samples were stored in air tight containers and maintained at 4°C.

The plant sample of *A. parviflora* was extracted using soxhlet extraction apparatus. Ten grams each of the powered sample was individually packed in muslin cloth and used for extraction at a temperature below the boiling temperature of each solvent, for 48 hours. Each 100 mL of solvents such as methanol, ethanol, chloroform, n-hexane, petroleum ether, ethyl acetate and acetone were used for the extractions. The extract was filtered through Whatman No.1 filter paper and concentrated using a rotary evaporator. The residue was dissolved in sterile DMSO (9:1) in 50 mg mL^-1^ concentration. The extract was filtered using 0.45 μ solvent-resistance filter (Millipore GV 0.45 μ) and stored at 4**°**C until use.

### Antimicrobial screening and susceptibility assay

The antimicrobial activity of the crude extracts of leaf was determined by the disc diffusion method [22] against the clinical microbial strains listed in Table 1. The test cultures maintained in nutrient agar slant at 4°C were sub-cultured in nutrient broth to obtain the working cultures approximately containing 10^6^ CFU mL^-1^.

**Table 1:** **Antimicrobial activity screening of *Artemisia parviflora *leaf extracts by disk diffusion method**

**Bacterial strains**	**Zone of inhibition (diameter in mm)**								
	**Positive control**	**Solvent control**	**Ethyl acetate**	**Ethanol**	**Methanol**	**Chloroform**	**N-Hexane**	**Petroleum ether**	**Acetone**
*Bacillus subtilis* (MTCC 441)	20	-	14 ± 0.0	12 ± 0.0	16 ± 0.0	14 ± 0.5	14 ± 0.0	10 ± 0.5	-
*Staphylococcus aureus* (MTCC 29212)	21	-	14 ± 0.1	12 ± 0.5	16 ± 0.5	15 ± 0.0	17 ± 0.0	-	-
*Escherichia coli* (ATCC 25922)	20	-	12 ± 0.5	13 ± 0.0	17 ± 0.5	14 ± 0.0	18 ± 1.0	9 ± 0.0	11 ± 0.0
*Yersinia enterocolitica* (MTCC 840)	17	-	15 ± 0.5	11 ± 0.5	15 ± 0.0	11 ± 0.5	15 ± 0.0	12 ± 0.0	13 ± 0.0
*Proteus vulgaris* (MTCC 1771)	16	-	12 ± 0.5	9 ± 0.0	13 ± 0.0	11 ± 0.5	13 ± 0.0	9 ± 1.0	11 ± 0.0
*Pseudomonas aeruginosa* (ATCC 27853)	21	-	14 ± 0.0	-	16 ± 0.0	-	18 ± 0.5	-	-
*Klebsiella pneumonia* (ATCC 15380)	18	-	13 ± 0.0	-	14 ± 0.0	10 ± 0.5	16 ± 0.5	-	-
*Shigella flexneri* (MTCC 1457)	20	-	14 ± 0.5	-	15 ± 0.5	13 ± 0.5	18 ± 0.0	12 ± 1.0	12 ± 0.0
*Enterococcus faecalis* (ATCC 29212)	21	-	14 ± 0.5	11 ± 0.0	13 ± 0.5	12 ± 1.0	14 ± 0.5	11 ± 0.0	12 ± 0.5
*Enterobacter aerogenes* (MTCC 111)	18	-	13 ± 0.5	12 ± 0.0	13 ± 0.5	11 ± 0.0	14 ± 1.0	11 ± 0.0	11 ± 1.0

± − mean standard deviation of triplicates, concentration of the extract 5 mg/dick. (−) No clear zone of inhibition was observed around the disk. Positive controls, ampicilin or streptomycin (10μg mL^-1^). Solvent control - 10% DMSO.

The extracts were incorporated in a 5 mm sterile disc at a concentration of 500 μg /disc. After complete saturation, the discs were dried overnight in a sterile chamber and used for screening for antimicrobial activity. Mueller Hinton (MH) agar plates were swabbed with each bacterial strain and the test disks were placed along with the control disks. Ampicillin or streptomycin disks (10 μg /disc) were taken as positive control and 10% dimethyl sulfoxide (DMSO) was taken as the negative solvent control. Plates were incubated overnight at 37°C. Clear, distinct zone of inhibition was visualized surrounding the discs. The antimicrobial activity of the test agents was determined by measuring the zone of inhibition expressed in mm.

The potential effectiveness of *A. parviflora* extracts was tested on all isolates by agar dilution susceptibility test. To determine the Minimum Inhibitory Concentration (MIC), test was performed based on modified method of NCCLS and CLSI [23,24]. Series of twofold dilutions were made to obtain a final concentration of the extracts in the agar medium ranging of 512 μg mL^-1^ to 16 μg mL^-1^. Pour plates were prepared and 2 μL of overnight grown cultures, approximately containing 1 × 10^4^ CFU mL^-1^, was spotted on solidified plates. The extract concentration showing ≥ 99% inhibition of visible colony growth is taken as the MIC for that organism.

### Phytochemical screening of constituents of *A. parviflora*

Standard phytochemical screening was performed to characterize the phytochemical constituents in the leaf extracts, [25,26]. Dragendorff’s and Meyer’s tests were used to test for alkaloids. The formation of reddish brown precipitate with Dragendorff’s reagent and cream with Mayer’s reagent was regarded as positive for the presence of alkaloids. Steroids were tested by Liebermann-Burchard test. Colour development form violet to blue or bluish-green indicate the presence of a steroid. Terpenoids were tested by Salkowski test, a reddish brown coloration of the interface indicates the presence of terpenoids. Copper acetate test was performed to identify the presence of diterpenes; formation of emerald green colour indicates the presence of diterpenes. Presence of triterpenoids was tested by Hirschhorn test; the change of yellow colour to red indicates the presence of triterpenoids. The formation of yellow colored layer in Salkowski test also indicated the presence of triterpenoids. Flavonoids are tested by FeCl_3_, lead ethanoate, Pew’s and Shinoda’s tests [27]. A green- blue or violet coloration by FeCl_3_, appearance of buff-coloured precipitate by lead ethanoate and Pink-tomato red coloration by Shinoda’s test indicated the presence of flavonoids in the extract.

Presence of tannins was checked by neutral ferric chloride and lead acetate tests, brownish green colour indicates the presence of tannins. The extract was treated with few drops of gelatin solution, the formation of white precipitate reveals the presence of phenols. Glycosides were tested by Fehling test. A deep red color which faded to brownish yellow indicates the presence of glycosides. Presence of saponins was tested by frothing test, frothing which persisted on warming was taken as an evidence for the presence of saponins. Borntrager’s test was performed to identify the presence of anthraquinones, appearance of a pink or violet colour in the lower phase was taken as the presence of free anthraquinones. The test for amino acids by ninhydrin, carbohydrates by Barfoed’s test, hydrolysable tannins, phlobatannins, resins, quinones and volatile oils were also carried out with little modification [26,28,29].

### Determination of total phenolic contents

The total phenolic content in *A. parviflora* extracts was determined using a modified Folin–Ciocalteu method [30,31]. 200 μL of sample was mixed with 2.6 mL of distilled water, 200 μL of Folin–Ciocalteu’s phenol reagent was added to each tube. The content was vortexed and incubated for 5 min. Then 2 mL of 7% Na_2_CO_3_ was added to each tube. The content in the tube was vortexed and incubated for 2 h with intermediate shaking. The absorbance of sample was read in spectrophotometer (Ultrospec 2100 pro, Amersham biosciences) at 725 nm. The total phenolic content is expressed as milligrams of Gallic acid per gram of dry extract.

### Antioxidant activity evaluation by ferric thiocyanate method

The antioxidant activity of *A. parviflora* leaf extracts were determined using FTC method [32]. Samples were dissolved in ethanol (1mg mL^-1^). To 1 mL of sample, 1mL of 2.5% linoleic acid in ethanol and 2 mL of 0.02 M phosphate buffer (pH 7.0) were added and the volume is made up to 5 mL using distilled water. The mixture was incubated at 40°C for 96 h. At every 12 h interval, 0.1 mL from each vial was diluted with 9.7 mL of 75% ethanol, 0.1 mL of 30% aqueous ammonium thiocyanate and 0.1 mL 0.02 M FeCl_2_ in 3.5% hydrochloric acid. After three minutes of incubation the absorbance of the resulting red colour was measured at 532 nm. Tert-butyl-1- hydroxyl toluene (BHT) was used as a positive control while the ethanol without sample was used as a negative control. The antioxidant activity was carried out in triplicates. The Percentage of lipid peroxidation inhibition was calculated by the following formula,

$$\text{Percentage}\;\text{of}\text{Lipid}\;\text{Peroxidation}\;\text{Inhibition}\;(\%LPI)=\frac{A_{532}\;\text{Control}\;-\;A_{532}\;\text{Sample}}{A_{532}\;\text{Sample}\;}X\;100$$

Where A_532_ control is the absorbance of the control and A_532_ Sample is the absorbance of sample.

### 2, 2-Diphenyl-1-Picrylhydrazyl (DPPH) scavenging activity

Hydrogen atom or electron donation ability was measured as the radical-scavenging activity. DPPH assay [33,34] was used to determine the radical scavenging activity of methanol and hexane leaf extract of *A. parviflora*. DPPH, a purple color solution, reacts with an antioxidant compound present in the test extracts, it is reduced to yield a light-yellow color diphenylpicrylhydrazyl which can be spectrophotometrically measured. To 4 mL of leaf extracts, 1 mL of 0.2 mM DPPH methanol solution was added. After incubating for 30 min in dark, the absorbance of the resulting solutions was measured at 517 nm using a spectrophotometer. Distilled water was used instead of sample as negative control. BHT and α-Tocopherol were used as positive controls. All the tests were carried out in triplicates. The Radical scavenging activity was calculated according to the equation:

$$\text{Percentage}\;\text{of}\;\text{Radicals}\;\text{Scavenging}\;\text{Activity}\;(\%RSA)=\;\frac{A_{517}\text{Control}\;-\;A_{517}\;\text{Sample}}{A_{517}\;\text{Sample}}\;X\;100$$

Where A_517_ control is the absorbance of the control and A_517_ Sample is the absorbance of samples.

### Statistical analysis

All analyses were done in triplicates in order to determine their reproducibility. The results were expressed as mean ± SD. Analysis of variance (ANOVA) was performed using Graph pad prism and Excel 2007. The probability value (p ≤ 0.05) was considered as significance.

## Results

The *A. parviflora* leaf extracts were tested for antimicrobial activity against ten clinically important microorganisms using agar dilution method. All the extracts of *A. parviflora* exhibited considerably broader inhibitory activity for the most of the test organisms (Table 1). High zone of inhibition was observed for n-hexane extract against *E. coli*, *S. flexneri* and *P. aeruginosa* (18 mm). The n-hexane, ethyl acetate and methanol extracts showed good activity compared to the other extracts. The minimum inhibitory concentration for n-hexane, ethyl acetate and methanol extracts of *A. parviflora* ranged from 32–64 μg mL^-1^ for all the microbes tested (Table 2). Growth of *P*. *aeruginosa* was inhibited by n-hexane (18 mm), methanol (16 mm) and ethyl acetate (14 mm) extracts with the MIC value of 32 μg mL^-1^.

**Table 2:** **Minimum Inhibitory Concentration (MIC) of various solvent extracts of *Artemisia parviflora *against test microorganisms**

**Bacterial strains**	**MIC μg mL**^**-1**^						
	**Ethyl acetate**	**Ethanol**	**Methanol**	**Chloroform**	**N- Hexane**	**Petroleum ether**	**Acetone**
*Bacillus subtilis*	32	64	32	32	32	64	128
*Staphylococcus aureus*	32	64	32	32	32	*	*
*Escherichia coli*	32	64	32	32	32	128	128
*Yersinia enterocolitica*	32	256	32	64	32	128	64
*Proteus vulgaris*	64	256	64	64	64	128	256
*Pseudomonas aeruginosa*	32	*	32	*	32	*	*
*Klebsiella pneumoniae*	64	*	64	128	34	*	*
*Shigella flexneri*	32	*	32	32	32	128	256
*Enterococcus faecalis*	32	256	64	64	32	128	128
*Enterobacter aerogenes*	32	64	64	128	32	128	256

* No inhibition was observed up to 512 μg mL^-1^.

Higher inhibition zone was observed with n-hexane (16mm) for *K. pneumoniae* while other extracts showed comparatively low inhibition zones. MIC of n-hexane extract was observed to be 32 μg mL^-1^ for *K. pneumoniae.* Ethyl acetate, methanol and chloroform extracts showed MIC in the range from 64–128 μg mL^-1^ for *K. pneumoniae.* Chloroform extract was equally effective against all the test microbes other than *P. aeruginosa*, with MIC ranging from 32–128 μg mL^-1^. Ethanol, petroleum ether and acetone extracts showed moderate antimicrobial activity against many test microbes but showed no antimicrobial activity against *P. aeruginosa* and *K. pneumoniae.* Ethanol extract was inactive for *S. flexneri* whereas moderate activity was observed for other test microbes with the MIC ranging from 64 to 256 μg mL^-1^. Petroleum ether and acetone extracts showed no inhibition against *S. aureus*. Acetone extract showed no inhibition for *B. subtilis.* MIC value for petroleum ether and acetone extract were higher (MIC > 64 μg mL^-1^) for the rest of the bacteria tested.

Phytochemical screening of *A. parviflora* extracts showed positive result for the alkaloids, steroids, terpenoids, di- and triterpenoids, flavonoids, tannins, phenols and volatile oils [Table 3. The n-hexane and methanol extracts from *A. parviflora* showed abundant terpenoids, diterpenoids, triterpenoids and volatile oil. Ethyl acetate extract showed abundant terpenoids. Tannins, hydrolysable tannins and phlobatannins were found in methanol, ethanol and n-hexane extracts. None of the extracts showed positive for glycosides, saponin, amino acids, carbohydrates, resins, quinones, and anthraquinones. Previous study had demonstrated that essential oil of *A. parviflora* is rich in terpenoids and steroids [9].

**Table 3:** **Phytochemical constituents of various solvent extracts of *Artemisia parviflora***

**Phytochemicals Screened**	**Extracts**						
	**Ethyl Acetate**	**Ethanol**	**Methanol**	**Chloroform**	**N- Hexane**	**Petroleum Ether**	**Acetone**
Alkaloids	+	++	++	++	+	+	+
Steroids	++	+	++	++	+	+	+
Terpenoids	++	+	++	+	++	+	+
Diterpenoids	++	+	++	+	++	-	-
Triterpenoids	++	+	++	+	++	-	-
Flavonoids	+	+	++	+	+	+	-
Tannins	-	+	++	-	+	-	-
Phenols	+	+	++	+	+	+	+
Glycosides	-	-	-	-	-	-	-
Saponin	-	-	-	-	-	-	-
Amino acid	-	-	-	-	-	-	-
Carbohydrates	-	-	-	-	-	-	-
Hydrolysable Tannins	-	-	+	-	+	-	-
Phlobatannins	-	-	+	-	-	-	-
Resins	-	-	-	-	-	-	-
Quinones	-	-	-	-	-	-	-
Volatile oils	+	+	++	++	++	+	+
Anthraquinones	-	-	-	-	-	-	-

(++) detected abundant, (+) detected, (−) not detected.

The total phenolic content of various extracts of *A. parviflora* is shown in Table 4. The total phenolic content of *A. parviflora* ranges from 37.18 to 15.30 mg mL^-1^ gallic acid equivalent (GAE) in 100 g of the dry sample. Methanol extract of A*. parviflora* contained high amount of phenolics (37.18 ± 3.78 mg mL^-1^ GAE) followed by ethyl acetate extract (23.633 ± 2.2 mg mL^-1^ GAE). Ethanol and n-Hexane extracts had relatively moderate total phenolic content of 19.307 ± 2.33 and 19.7422 ± 3.4 mg mL^-1^ GAE representatively. The phenolic content of chloroform, petroleum ether and acetone extracts ranged from 17.5–15.3 mg GAE. Equation of the regression line formula correlation coefficient were Y = 0.9004X + 0.1562 and r^2^ = 0.9940 with p <0.001.

**Table 4:** **Antioxidant, free radical scavenging activity and total phenolic content of various solvent extracts of *Artemisia parviflora***

**#8195;Extracts**	**Antioxidant activity**		**TPC mg mL**^**-1**^
	**DPPH Assay (%)**	**FTC (%)**	
BHT	80.00 ± 0.5	82.23 ± 0.5	NA
α-Tocopherol	65.70 ± 1.5	73.17 ± 0.2	NA
Ethanol	60.83 ± 1.6	60.04 ± 0.4	19.74 ± 3.40
Methanol	85.11 ± 1.0	78.42 ± 0.9	37.18 ± 3.78
Chloroform	55.27 ± 1.1	50.74 ± 0.2	17.44 ± 4.32
n-Hexane	57.14 ± 1.3	55.61 ± 0.3	19.30 ± 2.33
Acetone	45.99 ± 1.0	40.18 ± 0.3	15.30 ± 2.33
Petroleum ether	43.29 ± 1.0	49.20 ± 0.2	15.62 ± 1.80
Ethyl acetate	68.01 ± 0.5	61.51 ± 0.2	23.63 ± 2.20

± − mean standard deviation of triplicates, NA - not applicable. DPPH - 2, 2-Diphenyl-1-Picrylhydrazyl, FTC - ferric thiocyanate, TPC- Total Phenolic content, BHT- Tert-butyl-1- hydroxyl toluene.

The antioxidant activity of *A. parviflora* extracts was determined by the peroxidation of linoleic acid emulsion using ferric thiocyanate method. Low absorbance values in the FTC method indicate a high level of antioxidant activity during emulsion incubation. Peroxide formation during emulsion was compared with BHT and α-tocopherol as standard samples. BHT and α-tocopherol (50 μg mL^-1^) inhibited 82.6% and 73% peroxidation of linoleic acid emulsion respectively. Methanol extract showed a significantly strong antioxidant activity (78.42 ± 0.9). Ethyl acetate, ethanol, hexane and chloroform extracts of *A. parviflora* were less effective than the methanol extract and the activity ranged from 61.51 ± 0.2 to 50.74 ± 0.2%. Though these extracts contained less abundant of flavonoids and tannins, they showed moderate antioxidant activity. The petroleum ether and acetone extracts showed less antioxidant activity (49.20 ± 0.2 and 40.18 ± 0.3%).

The free radical scavenging activity of *A. parviflora* extracts was assessed by the DPPH assay. DPPH is a stable nitrogen centered free radical which can be effectively scavenged by antioxidants. Methanol extract showed a high percentage of DPPH scavenging activity of 85.11 ± 1.0% followed by ethyl acetate (68.01 ± 0.5%), ethanol (60.83 ± 1.6%), n-hexane (57.14 ± 1.3%) and chloroform (55.27 ± 1.1%) extracts. Acetone and petroleum ether extracts showed least DPPH scavenging activity of 45.99 ± 1.0% and 43.29 ± 1.0% respectively.

## Discussion

The major components the essential oil of *Artemisia* spp. were of phenolics, flavonoids and tannins (19). The various degrees of microbial inhibitory effects of essential oil of *A. absinthium*, *A. dracunculus*, *A. santonicum*, and *A. spicigera* have been reported [10]. Though the essential oil of these species showed good antimicrobial activity they posses weak antioxidant activity [17,18]. In the present study, n-hexane extract of *A. parviflora* showed high level of antimicrobial activity for all the microbes tested with the MIC value of 32–64 mg mL^-1^. Methanol, ethyl acetate and chloroform extracts of *A. parviflora* showed moderate antimicrobial activity compared to the ethanol, petroleum ether and acetone extracts. Growth of *P. aeruginosa* was inhibited by the n-hexane, ethyl acetate and methanol extracts compared to the antibiotics standards used.

The high microbial activity of n-hexane, ethyl acetate and methanol extracts could be attributed to the higher concentration of active antimicrobial agents like terpenoids, phenolics and volatile oils, in *Artemisia* species [35,36]. Good antimicrobial activity observed for n-hexane, methanol and ethyl acetate extracts of *A. parviflora* also could be due to the presence of terpenoids present. Terpenoids are one of the most abundant active antimicrobial agents isolated from plants [37,38]. The antibacterial activity of the terpenoids is mostly dependant on the interaction with the cell membrane of microorganisms. Terpenoids compounds interact with the lipid components of cell membranes, easily penetrate into the interior of the cell and kill the cells by interacting with the intracellular material [39-41].

In our study, n-hexane and methanol extracts showed good level of inhibition for *P. aeruginosa, E. coli, K. pneumoniae, S. flexneri* and *B. subtilis.* The phytochemical assay showed that the methanol extract of *A. parviflora* was abundant in phenolics. *A. parviflora* extracts can be also considered as a good source of phenolic substances like phenolic acids and tannins with a potential medicinal use. The active compounds present in these extracts may serve as good alternative for the conventional antibiotics drugs to treat multidrug resistance.

Phenolic compounds from plant extracts may contribute directly to antioxidant activity. Methanol extract of *A. parviflora* possesses high antioxidant activity. The total phenolics content of the *A. parviflora* was significantly high in methanol extract. The antioxidant activity of extract could be due to the presence of high content of phenolics, flavonoids and tannins. Although, *A. parviflora* extracts had low content of tannins and flavonoids, they showed good antioxidant activity. The n-hexane extract showed high antimicrobial activity but only showed moderate free radical scavenging activity with DPPH and FTC revealing that the active compounds found in the n-hexane showed less antioxidant activity when compared to methanol extract of *A. parviflora*. The active components of *A. parviflora* might be helpful in preventing or slowing the progress of various oxidative stress related disorders.

## Conclusions

This study presents the antimicrobial activity against reference bacterial strains that are implicated as opportunistic as well as nosocomial infections, antioxidant activity and phytochemical components of the solvent extracts of *A. parviflora*. Further investigation is necessary to separate the active components and evaluate the antioxidant and antimicrobial activity of each component, so as to confirm the bioactive nature of these compounds.

## Competing interests

The authors declared no potential conflicts of interests with respect to the authorship and/or publication of this article.

## Authors’ contributions

ARA performed the analyses, interpreted the results and prepared the manuscript. WH supervised the work, evaluated the results and corrected the manuscript for publication. Authors read and approved the final manuscript.
